# *Vibrio vulnificus* outer membrane vesicles induce mitochondrial dysfunction in macrophages via the TNF signaling pathway

**DOI:** 10.3389/fvets.2026.1785223

**Published:** 2026-05-11

**Authors:** Jun Li, Luying Wang, Yafang Zhou, Lijie Fan, Yongliang Lou, Xingxing Xiao, Shuai Gao

**Affiliations:** Key Laboratory of Laboratory Medicine, Ministry of Education, School of Laboratory Medicine and Life Sciences, Wenzhou Medical University, Wenzhou, China

**Keywords:** macrophages, mitochondrial dysfunction, outer membrane vesicles (OMVs), TNF-α, *Vibrio vulnificus*

## Abstract

*Vibrio vulnificus* (*V. vulnificus*) is a highly virulent zoonotic pathogen causing severe septicemia and tissue necrosis in humans, characterized by a cytokine storm. While bacterial outer membrane vesicles (OMVs) are known to mediate host-pathogen interactions, their specific contribution to *V. vulnificus*-induced immunopathology remains obscure. In this study, we isolated and characterized *V. vulnificus*-derived OMVs and investigated their impact on murine macrophages (J774A.1). We demonstrate that these OMVs are actively internalized by macrophages in a time-dependent manner, triggering a robust pro-inflammatory response. Transcriptomic analysis and subsequent validation revealed that internalized OMVs activated the TNF signaling pathway, leading to the phosphorylation of NF-κB and MAPK (p38/JNK) cascades. Crucially, OMV exposure induced severe mitochondrial dysfunction, evidenced by ultrastructural disruption and a significant reduction in mitochondrial membrane potential. Notably, pharmacological blockade of the TNF receptor with the antagonist R-7050 effectively attenuated this mitochondrial damage. These findings establish a novel pathogenic mechanism wherein *V. vulnificus* OMVs exploit the host TNF signaling axis to drive mitochondrial injury, providing new insights into the cellular mechanisms of *V. vulnificus* lethality and suggesting the TNF-mitochondria axis as a potential therapeutic target.

## Introduction

*Vibrio vulnificus* (*V. vulnificus*) is a highly virulent, Gram-negative bacterium that poses a significant threat to public health, particularly in coastal regions where seafood consumption and exposure to seawater are common (1). This pathogen is responsible for a range of severe infections, including primary septicemia, necrotizing wound infections, and gastroenteritis (2). The case-fatality rates for these infections are alarmingly high, with primary septicemia exceeding 50% and wound infections affecting approximately 15% of patients (3, 4). The bacterium's ability to cause such severe disease is attributed to its numerous virulence factors, including the production of cytotoxins, capsular polysaccharides, and the multifunctional autoprocessing repeats-in-toxin (MARTX) toxin, all of which play crucial roles in immune evasion and tissue destruction (5, 6).

One of the critical aspects of *V. vulnificus* infection is its interaction with macrophages, which are key players in the host's immune defense. Macrophages are not only involved in the initial immune response but also in the regulation of inflammation and tissue repair. The interaction between *V. vulnificus* and macrophages is complex and involves several molecular pathways that can either enhance or suppress the host's immune response. Macrophage migration inhibitory factor (MIF) is one of the key regulators of the inflammatory response during *V. vulnificus* infection. MIF facilitates the activation of nuclear factor-kappa B (NF-κB), which in turn upregulates the production of pro-inflammatory cytokines such as interleukin-6 (IL-6) (7). This pro-inflammatory response is crucial for controlling the infection but can also lead to tissue damage if not properly regulated. Additionally, the NLRP3 inflammasome is another critical component in macrophages that modulates the immune response to *V. vulnificus*. NLRP3 is involved in the metabolic reprogramming of macrophages, enhancing glycolysis and Reactive Oxygen Species (ROS) production, which are essential for an effective immune response (8).

TNF-α is a key mediator of inflammation and is involved in the activation of various immune cells, including macrophages and dendritic cells, which are crucial for mounting an effective immune response against bacterial infections (9). The interaction of TNF-α with macrophages during mycobacterial infections is essential for maintaining granuloma formation, a key feature of the host's defense against mycobacteria (10). TNF and its receptors TNFR1 and TNFR2 are tightly regulated to balance pro-inflammatory and anti-inflammatory responses in mycobacterial infections (11). This regulation is crucial for effective immune responses and highlights potential therapeutic targets for enhancing host defense mechanisms against mycobacterial infections. The role of TNF-α in *V. vulnificus* infections is underscored by its association with increased mortality rates in septic patients (12). Elevated serum levels of TNF-α have been observed in non-survivors compared to survivors, suggesting its potential as an early biomarker for predicting patient outcomes (12). Despite its clinical importance, the role of TNF-a during *V. vulnificus* infection is still not fully understood.

Bacterial outer membrane vesicles (OMVs) are small, spherical structures released from the outer membrane of Gram-negative bacteria (13). These vesicles play a crucial role in bacterial physiology and pathogenesis, acting as vehicles for the delivery of biomolecules such as proteins, lipids, and nucleic acids to host cells (14). OMVs have been shown to facilitate bacterial survival under stress conditions, mediate host-pathogen interactions, and contribute to the modulation of host immune responses (15). A critical aspect of their pathogenicity is their ability to induce mitochondrial damage, which is a key factor in the virulence of certain bacterial infections. *Acinetobacter baumannii*, a highly antibiotic-resistant pathogen, releases OMVs containing the outer membrane protein A (OmpAAb). These OMVs are internalized by host cells, where OmpAAb activates the host GTPase dynamin-related protein 1 (DRP1), leading to mitochondrial fragmentation (16). This mitochondrial disruption is a pivotal factor in the pathogenesis and dissemination of *A. baumannii* infections, as demonstrated in both *in vitro* and *in vivo* models. *Neisseria gonorrhoeae* utilizes OMVs to deliver the porin protein PorB to host mitochondria, triggering apoptosis in macrophages, which collectively contribute to the bacterium's ability to evade the host's innate immune response and promote infection (17). These findings underscore a growing paradigm where pathogens utilize OMVs to target mitochondria, thereby triggering host damage.

In this study, we demonstrate that *V. vulnificus*-derived OMVs are internalized by macrophages and induce the robust secretion of TNF-α and the activation of the TNF signaling pathway. Furthermore, we provide evidence that TNF signaling pathway acts as a critical upstream signal, triggering mitochondrial structural and functional damage, thereby elucidating a novel mechanism of *V. vulnificus*-induced host cell damage.

## Materials and methods

### Cell culture and bacterial strains

J774A.1 cells were sourced from the Cell Bank of Type Culture Collection of Chinese Academy of Sciences in Shanghai. Cells were cultured in RPMI 1640 medium supplemented with 10% fetal bovine serum (Gibco) under conditions of 37°C with 5% CO_2_.

*Vibrio vulnificus* ATCC 27562 strain was stored at −80 °C in our laboratory. Prior to use, the frozen stock was revived on Brain Heart Infusion Broth (BHI) agar plates, and incubated at 37 °C for 18 h.

### OMV extraction and characterization

To isolate *Vibrio vulnificus* OMVs, bacteria were cultured in BHI broth to stationary phase, and then the supernatant was collected via centrifuge at 8,000 × g for 10 min. The supernatant was filtered through a 0.22 μm filter (JET BIOFIL) and subsequently centrifuged at 200,000 × g for 2 h to obtain OMV pellets. The obtained OMVs were resuspended in PBS, and their protein content was quantified using the BCA Protein Assay Kit (Beyotime).

### Confocal microscopy

2 × 10^5^ J774A.1 cells were seeded onto glass coverslips (WHB Scientific) within a 12-well plate. Following a 12-hour attachment culture period, DiO-OMVs at a concentration of 2 μg/ml were introduced and incubated at 37 °C with 5% CO_2_ for durations of 2, 4, or 8 h, respectively. Subsequently, the cells were fixed using ice-cold methanol for 20 min, followed by the treatment with QuickBlock blocking buffer (Beyotime) for 20 min. Nuclear staining was conducted with a 10 μg/ml DAPI solution (Beyotime) at room temperature for 5 min. To assess the mitochondria membrane potential, 100 mm TMRM (Thermo Fisher) was incubated with cells seeded in glass bottom cell culture dishes (NEST) for 30 min. Image acquisition was performed using a confocal microscope (Nikon).

### Transcriptome analysis

2 × 10^5^ J774A.1 cells were treated with 2 μg/ml of OMVs for 4 h, and then total RNA was extracted from cellular samples using Trizol (Invitrogen). Library preparation was performed following mRNA enrichment, cDNA synthesis, end repair, A-tailing, adapter ligation, and PCR amplification. The cDNA libraries were sequenced on the MGI sequencing platform by Metware Biotechnology Co., Ltd. (Wuhan, China). Differential expression analysis between the OMV-treated and PBS control groups was conducted using DESeq2 (v1.38.3), with genes meeting the thresholds of |Log2FoldChange| > 0.263 and adjusted *p*-value < 0.05 considered significantly differentially expressed.

### ELISA

TNF-α secretion in J774A.1 cell culture supernatants was quantified using an ELISA kit (ABclonal), following the manufacturer's instructions. Briefly, samples were incubated in pre-coated wells, followed by the sequential addition of detection antibodies and HRP-streptavidin. Absorbance was measured at 450 nm after TMB development, and concentrations were calculated using a standard curve.

### Western blot and Coomassie Brilliant Blue staining

The samples were subjected to boiling at 95 °C for 10 min in loading buffer (Beyotime) prior to undergoing SDS-polyacrylamide gel electrophoresis. Proteins were then transferred onto polyvinylidene difluoride membranes (Bio-Rad) using a constant current of 300 mA for 90 min. The membranes were subsequently blocked with 5% (w/v) bovine serum albumin for 2 h, followed by incubation with primary antibodies diluted in Western blot Primary Antibody Dilution Buffer (Beyotime). The primary antibodies applied in this study included rabbit anti-NF-κB monoclonal antibodies (Beyotime, 1:500), rabbit anti-p-NF-κB monoclonal antibodies (CST, 1:1,000), rabbit anti-p38 monoclonal antibodies (Beyotime, 1:500), rabbit anti-p-p38 monoclonal antibodies (CST, 1:1,000), rabbit anti-JNK monoclonal antibodies (Beyotime, 1:500), rabbit anti-p-JNK monoclonal antibodies (Beyotime, 1:500), rabbit anti-GAPDH monoclonal antibodies (Beyotime, 1:1,000). Thereafter, the membrane was incubated with horseradish peroxidase (HRP)-conjugated goat anti-rabbit IgG antibody (Beyotime, 1:1,000) or HRP-conjugated goat anti-mouse IgG antibody (Beyotime, 1:1,000). The protein bands were visualized utilizing the Enhanced Chemiluminescence Western Blotting Substrate (GE).

For Coomassie Brilliant Blue staining, the gel was stained with Coomassie Brilliant Blue staining solution (Solarbio) for 1 h, followed by overnight washing with decolorising solution.

### Transmission electron microscopy

J774A.1 cells pretreated with or without R-7050 (Selleck, 1 μm) were stimulated with 2 μg/ml of OMVs, and then were fixed with 2.5% glutaraldehyde for 12 h. The samples were incubated with 1% osmium tetroxide for 1 h, then stained with 2% uranyl acetate for 1 h, followed by dehydration with acetone and embedding in epoxy resin. The prepared sections were stained with uranyl acetate and lead citrate, and then were visualized using a Hitachi transmission electron microscope.

To evaluate the morphology of the *Vibrio vulnificus* OMVs, 10 μl aliquot of the sample was applied to a carbon coated grid that had been glow discharged for 1 min in air, and the grids were immediately negatively stained using 2%phosphotungstic acid for 60 s.

### Quantification and statistics

All quantitative data represent at least three independent biological replicates. For confocal microscopy, a minimum of 50 images were analyzed per group. Statistical significance was determined using one-way ANOVA analysis with GraphPad Prism 9 software.

## Results

### Isolation and characterization of *V. vulnificus* OMVs

**Figure 1:**
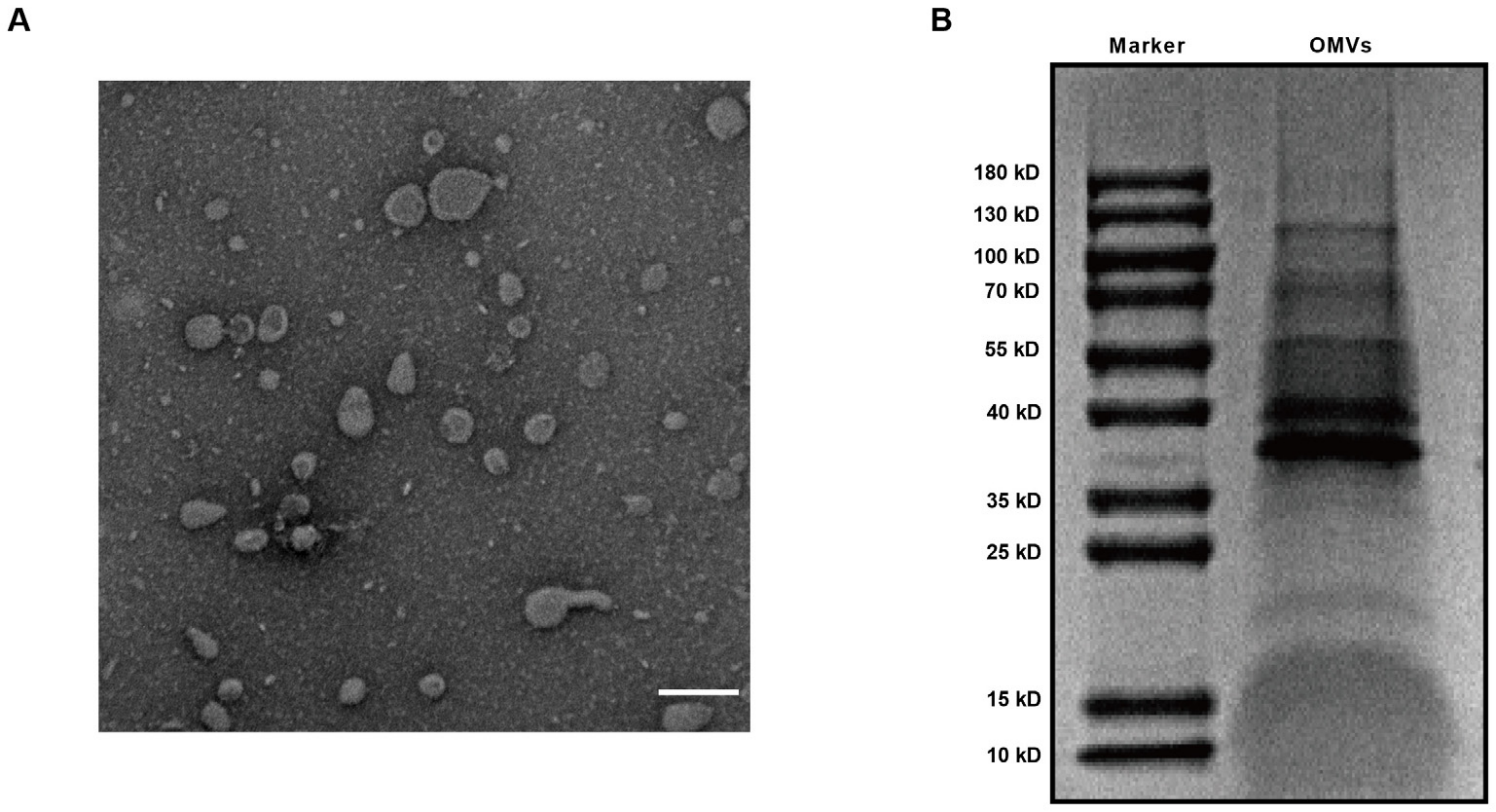
Characterization of *V. vulnificus*-derived OMVs. **(A)** Representative TEM image of purified OMVs. Scale bar: 100 nm. **(B)** Protein profiling of *V. vulnificus* OMVs using SDS-PAGE analysis, followed by staining with Coomassie Brilliant Blue.

To delve into the pathogenic mechanisms and biological significance of OMVs secreted by *V. vulnificus*, we initiated our study by isolating these vesicles from the cell-free culture supernatant using a standardized and rigorous ultracentrifugation method. Ensuring the structural integrity and purity of the OMVs is a fundamental prerequisite for all subsequent functional assays. Transmission electron microscopy (TEM) was employed to visualize the ultrastructure of the purified OMVs. The TEM micrographs revealed that the *V. vulnificus*-derived OMVs predominantly exhibited a characteristic spherical, closed-loop, bilayered membrane structure, with diameters ranging from 40 to 150 nm (Figure 1A). These vesicles maintained high structural fidelity, with no discernible contamination from flagellar fragments or other bacterial cellular debris, indicating a high degree of purity (Figure 1A).

Furthermore, we characterized the protein profiling of these vesicles to confirm their cargo complexity. SDS-PAGE, followed by Coomassie Brilliant Blue staining, showed that the isolated OMVs contained a diverse and sophisticated array of protein components. The electrophoretic pattern was characterized by multiple distinct and sharp bands, mainly ranging from approximately 35 kD−130 kD (Figure 1B). Significantly, the prominent bands observed in the 37–40 kDa range are likely indicative of major outer membrane proteins (MOMPs), such as OmpU and OmpA, which are recognized as predominant constituents of the *V. vulnificus* outer membrane. Collectively, these comprehensive characterizations confirm the successful isolation of high-quality, protein-rich *V. vulnificus* OMVs, providing a robust experimental foundation for investigating their interactions with host immune cells.

### Dynamic internalization of *V. vulnificus* OMVs by J774A.1 cells exhibits a time-dependent manner

The capacity of bacterial OMVs to engage with and penetrate host cells is a critical step in their ability to modulate host physiological and pathological responses. To investigate whether *V. vulnificus* OMVs can be actively internalized by macrophages, we utilized the green lipophilic fluorescent probe DiO (33'-dioctadecyloxacarbocyanine perchlorate) to label the lipid membranes of the OMVs. These labeled vesicles were then co-incubated with J774A.1 cells, and the dynamic process of cellular uptake was monitored using confocal microscopy at various time intervals (0, 2, 4, and 8 h).

**Figure 2:**
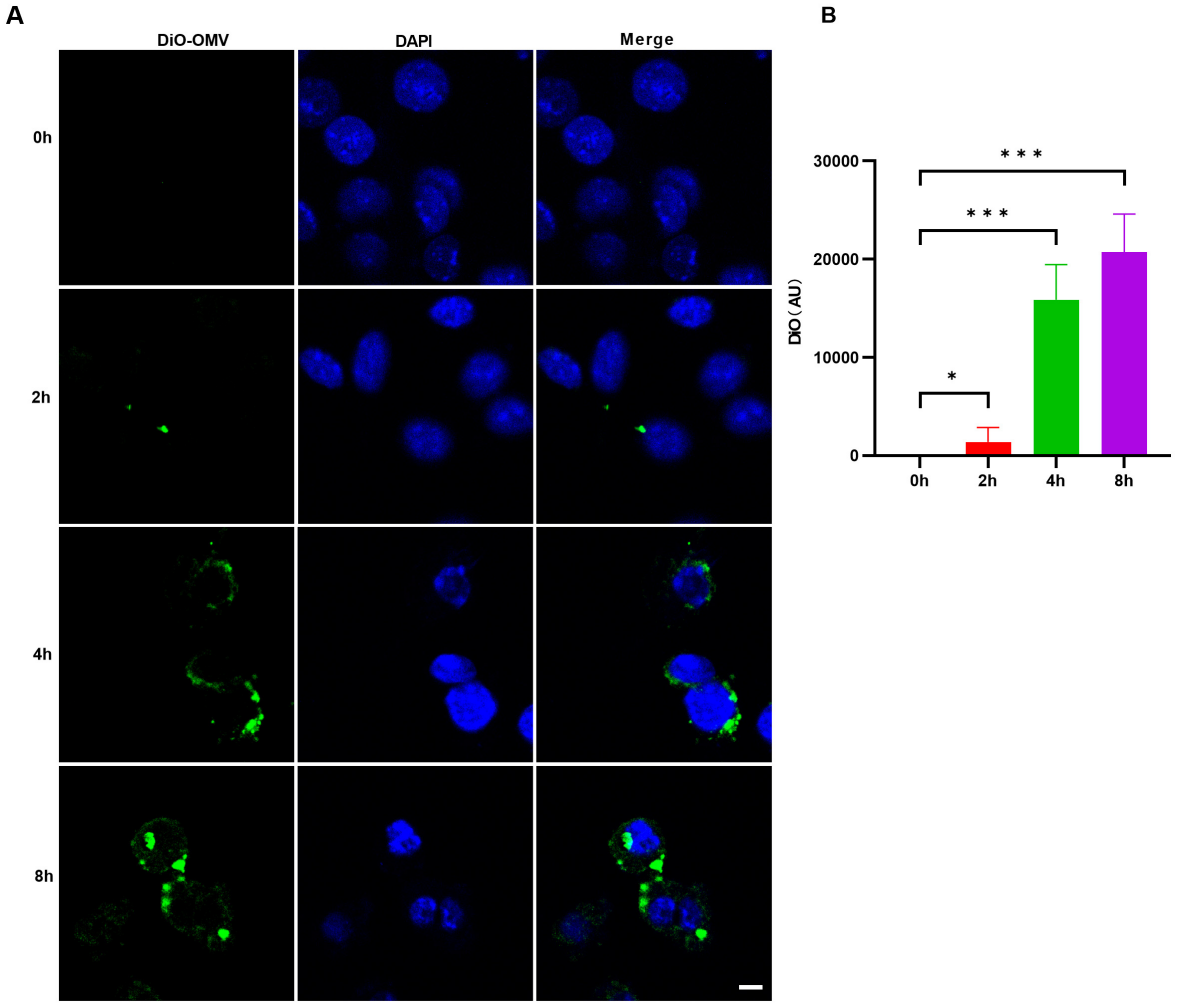
Time-dependent internalization of *V. vulnificus* OMVs by J774A.1 macrophages. **(A)** Representative confocal microscopy images of J774A.1 cells incubated with DiO-labeled OMVs for 0, 2, 4, and 8 h. Nuclei were stained with DAPI. Scale bar: 5 μm. **(B)** Quantitative analysis of the internalization of DiO-labeled OMVs using fluorescence intensity measurement. The results are presented as the means ± s.d.; *n* = 50 images. Significant differences compared to the 0-h time point were determined using a one-way ANOVA followed by Dunnett's multiple comparisons test. *P < 0.05, ***P < 0.001.

At the initial time point (0 h), no significant intracellular green fluorescence was detected (Figures 2A, B). However, after a 2-h incubation period, faint but discernible green fluorescent puncta began to emerge within the cytoplasmic region of the macrophages, signaling the commencement of OMV internalization. As the incubation time progressed to 4 and 8 h, there was a profound and progressive escalation in the intracellular fluorescence intensity (Figures 2A, B). Confocal microscopy imaging revealed that the internalized OMVs were not uniformly distributed but rather formed aggregated clusters, and a part of them was localized in the perinuclear region of the cytoplasm (Figure 2A). The observed time-dependent increase in fluorescence signals suggests that these vesicles serve as effective carriers for delivering bacterial components into the intracellular environment of host macrophages. These observations provide compelling visual evidence that J774A.1 cells are capable of actively and efficiently internalizing *V. vulnificus* OMVs.

### *V. vulnificus* OMVs orchestrate the activation of the TNF signaling pathway and downstream inflammatory cascades

**Figure 3:**
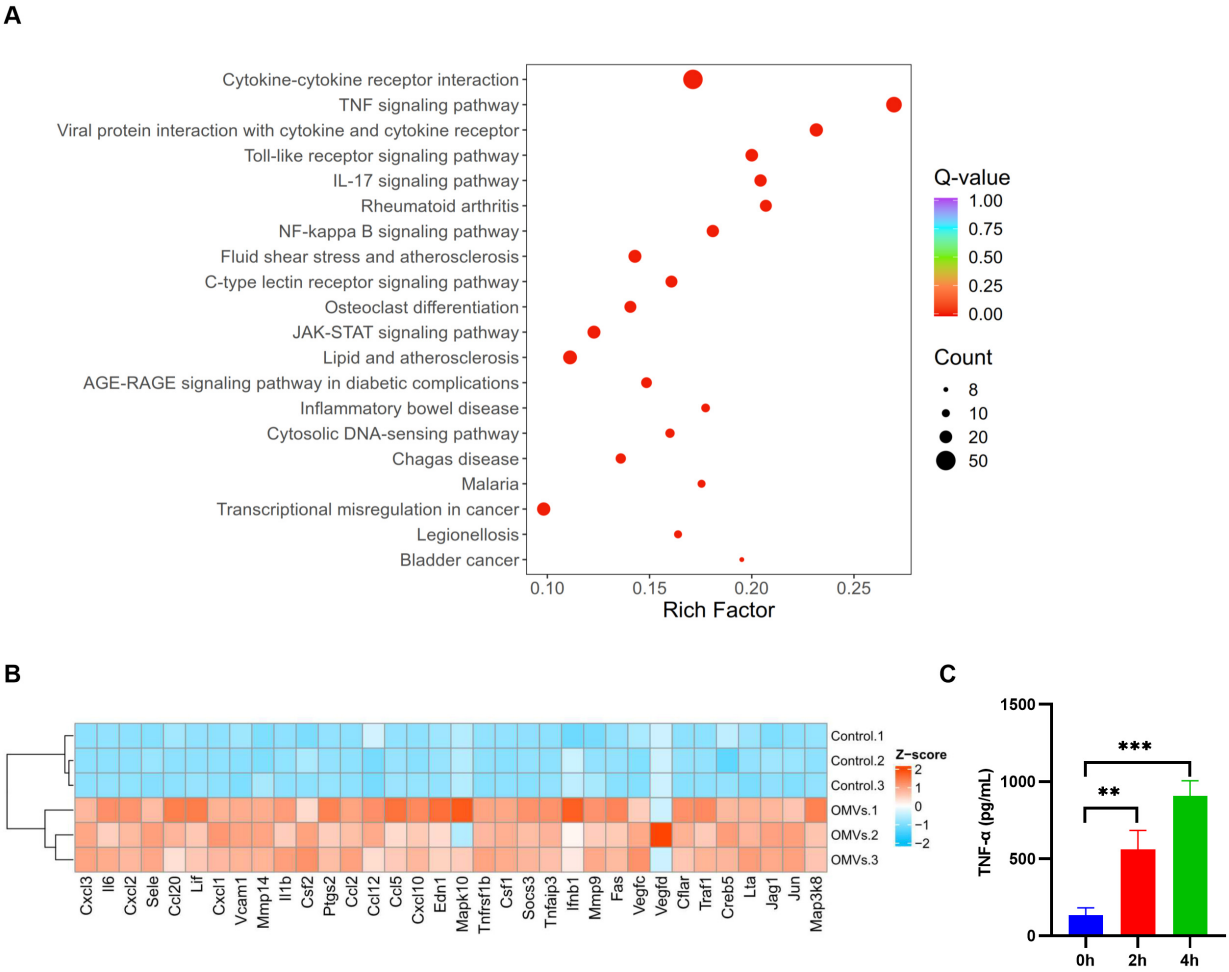
*V. vulnificus* OMVs induce the activation of TNF signaling pathway. **(A)** KEGG pathway enrichment analysis of differentially expressed genes (DEGs) in J774A.1 cells following OMV stimulation for 4 h. **(B)** Heatmap of differentially expressed genes enriched in the TNF signaling pathway identified by KEGG analysis. **(C)** The secretion of TNF-α by J774A.1 cells following exposure to *V. vulnificus* OMVs over 0–4 hour period in culture supernatants was quantified using ELISA. The data are presented as the means ± s.d.; *n* = 3. Significant differences compared with 0 h were identified by one-way ANOVA followed by Dunnett's multiple comparisons test. **P < 0.01, ***P < 0.001.

To elucidate the macrophage response to OMV stimulation, we performed transcriptome sequencing (RNA-seq) on J774A.1 cells treated with *V. vulnificus* OMVs for 4 h. Subsequent KEGG (Kyoto Encyclopedia of Genes and Genomes) pathway enrichment analysis of the differentially expressed genes revealed a profound activation of several core pro-inflammatory pathways. Notably, the TNF signaling pathway emerged as one of the most significantly enriched pathways (Figure 3A). Heatmap of differentially expressed genes enriched in the TNF signaling pathway demonstrated that most genes were up-regulated upon OMVs treatment (Figure 3B), including key pro-inflammatory chemokines (Cxcl3, Cxcl2, Cxcl1, Cxcl10, Ccl20, Ccl2, Ccl12, Ccl5), cytokines (Il6, Il1b, Lif), the TNF receptor superfamily member (Tnfrsf1b), MAPK signaling components (Mapk10, Map3k8), transcription factors (Jun, Creb5), and negative regulators of inflammation (Tnfaip3, Socs3). Concurrently, the enrichment of cytokine-cytokine receptor interaction and IL-17 signaling pathways underscored a robust inflammatory mobilization (Figure 3A). This comprehensive transcriptomic signature indicates that these interconnected signaling hubs, particularly the TNF axis, serve as the drivers of the macrophage response to OMV challenge.

**Figure 4:**
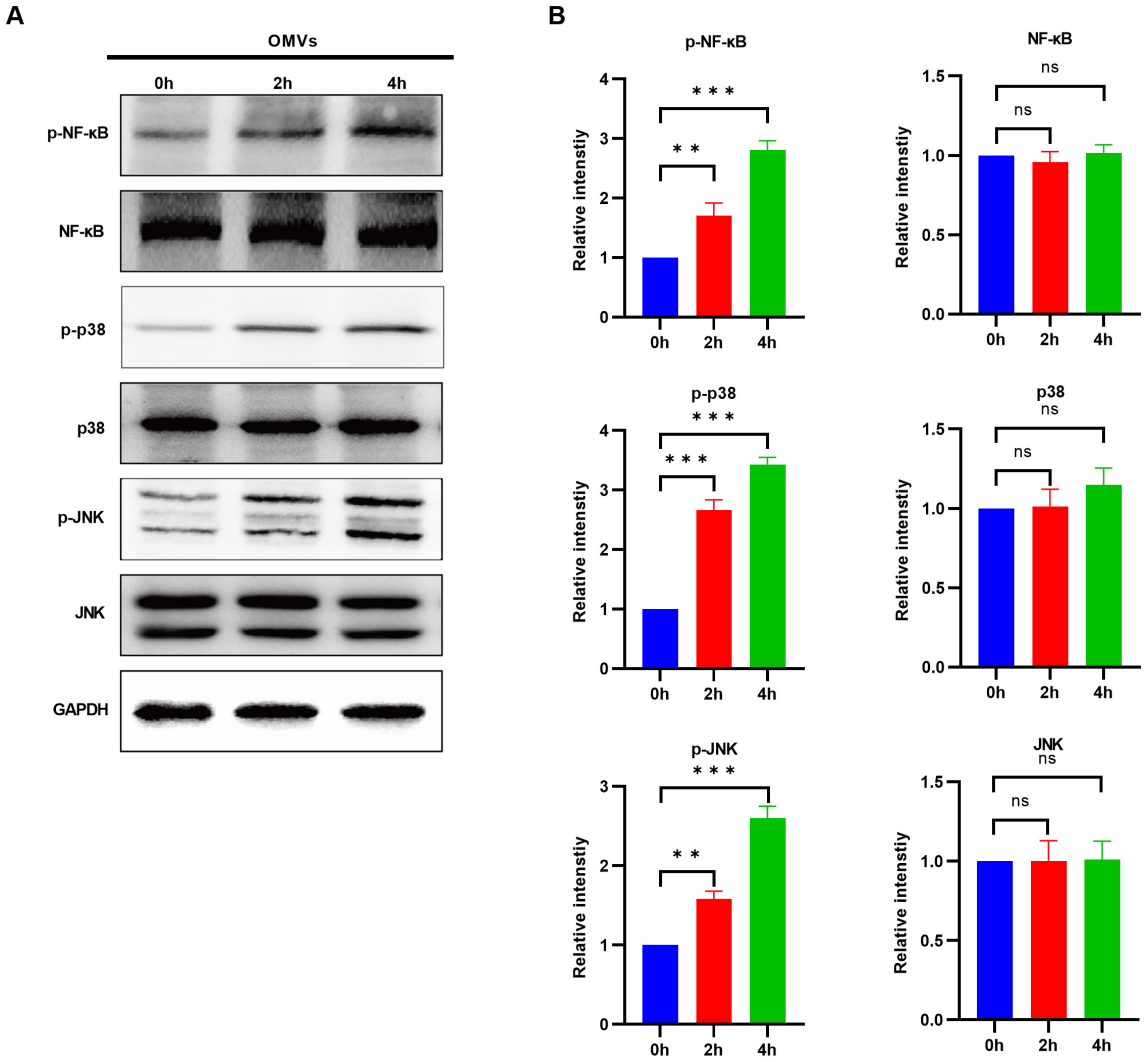
*V. vulnificus* OMVs activate NF-κB and MAPK signaling pathways. **(A)** Western blot analysis of the total and phosphorylated levels of NF-κB, p38, and JNK in J774A.1 cells at 0, 2, and 4 h following OMV stimulation. GAPDH served as the loading control. **(B)** Quantification of the ratios of phosphorylated to total protein in panel A. The data are presented as the means ± s.d.; *n* = 3. Significant differences compared with the 0 h group were identified via one-way ANOVA followed by Dunnett's multiple comparisons test. **P < 0.01, ***P < 0.001.

Further analysis demonstrated that the level of TNF-α secreted by J774A.1 cells was significantly increased with prolonged OMV stimulation time (Figure 3C). Considering that TNF-α functions as a master regulator, capable of activating multiple intracellular signaling cascades through receptor binding, we conducted an in-depth analysis of its downstream effectors. We assessed the activation status of its core downstream inflammatory pathways, specifically NF-κB, p38, and JNK, utilizing Western Blot analysis. The results showed that, compared to the untreated group (0 h), the protein levels of p-NF-κB, p-p38, and p-JNK were significantly elevated after 2 and 4 h of OMV stimulation, indicating a distinct phosphorylation activation, whereas levels of total target protein remained unchanged (Figures 4A, B). Collectively, these results demonstrate that following cellular entry, *V. vulnificus* OMVs induce secretion of TNF-α, which triggers the phosphorylation cascade of downstream NF-κB and MAPK (p38/JNK) pathways, thereby initiating an inflammatory response.

### TNF signaling pathway mediates *V. vulnificus* OMV-induced mitochondrial damage

**Figure 5:**
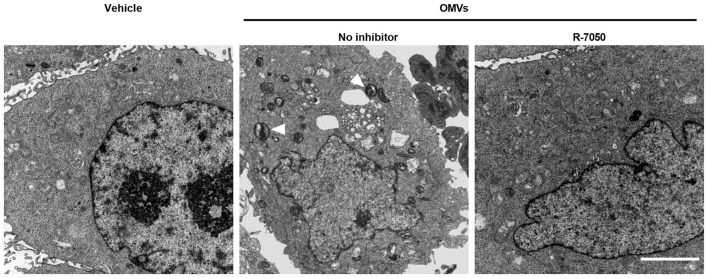
TNF signaling pathway contributes to OMV-induced mitochondrial ultrastructural damage. TEM images showing mitochondrial morphology in J774A.1 cells stimulated with *V. vulnificus* OMVs for 4 h with or without R-7050 treatment. Scale bar: 5 μm.

**Figure 6:**
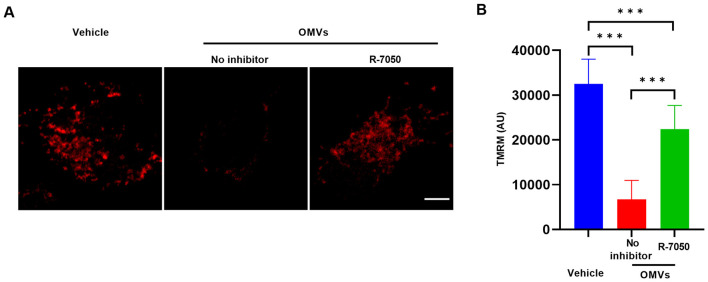
TNF signaling pathway mediates the dissipation of mitochondrial membrane potential. **(A)** Representative confocal images of TMRM-stained J774A.1 cells stimulated with *V. vulnificus* OMVs for 4 h with or without R-7050 treatment. Scale bar: 5 μm. **(B)** Quantitative analysis of the fluorescence intensity of TMRM in **(A)**. The data are presented as the means ± s.d.; *n* = 50 images. Significant differences compared with the vehicle-treated group were identified by one-way ANOVA followed by Dunnett's multiple comparisons test. ***P < 0.001.

Extensive inflammatory activation is frequently accompanied by disrupted mitochondrial homeostasis (18), and we hypothesized that the OMV-induced TNF-α surge might be a key factor contributing to mitochondrial impairment. To establish a definitive causal link between the TNF signaling axis and mitochondrial homeostasis, we utilized the TNF-α receptor antagonist R-7050 to perform blockade experiments. We examined the mitochondrial ultrastructure using TEM. In the vehicle-treated group, J774A.1 cells displayed healthy mitochondria characterized by intact double membranes and densely packed, well-organized cristae (Figure 5). In stark contrast, macrophages challenged with *V. vulnificus* OMVs exhibited profound and widespread mitochondrial pathology. These alterations included significant mitochondrial swelling, a marked loss of cristae density, and the presence of widespread vacuolization within the mitochondrial matrix (Figure 5). Remarkably, pretreatment of the cells with the R-7050 resulted in a significant mitigation of these ultrastructural defects. The mitochondria in the R-7050-treated group maintained near-normal morphology, with preserved cristae architecture and reduced swelling, highlighting the protective effect of TNF signaling inhibition. To further evaluate the detrimental effects of OMVs on mitochondrial function and the role of the TNF pathway in this process, we assessed changes in mitochondrial membrane potential (MMP) using TMRM staining method. The results demonstrated that compared with the control group, the TMRM fluorescence intensity was significantly reduced in the OMV-treated group (Figures 6A, B). In contrast, pharmacological inhibition of the TNF pathway with R-7050 effectively mitigated this loss. These results suggest that *V. vulnificus* OMVs trigger mitochondrial structural and functional impairment through the activation of the TNF-mediated signaling cascade.

## Discussion

Understanding the mechanisms by which *Vibrio vulnificus*, a highly virulent and rapidly invasive pathogen, compromises host immune defenses is of paramount importance. The role of OMVs as pivotal mediators in host-pathogen interactions, transcending the physical confines of the bacterium, is gaining increasing recognition. This study reveals a novel mechanism whereby OMVs secreted by *V. vulnificus* induce inflammatory pathology. Our findings demonstrate that these OMVs are internalized by J774A.1 cells, eliciting a pronounced pro-inflammatory response characterized by elevated secretion of TNF-α and activation of the TNF signaling pathway. Importantly, we provide compelling evidence that OMVs directly cause mitochondrial damage, evidenced by ultrastructural condensation and the loss of MMP. This mitochondrial damage is mechanistically dependent on TNF signaling, as it is significantly attenuated by the specific TNFR inhibitor R-7050. These findings position OMVs of *V. vulnificus* as central orchestrators of a TNF-mediated mitochondrial injury cascade within macrophages, which may be pivotal to the hyperinflammatory state and tissue damage observed in *V. vulnificus* infections.

Our initial observation that *V. vulnificus* OMVs are efficiently internalized by J774A.1 macrophages aligns with the established capacity of OMVs from various Gram-negative bacteria to enter host cells via endocytosis or phagocytosis (19–21). This internalization is the critical first step, delivering a concentrated payload of virulence factors, including possible hemolysins, proteases, and LPS, directly into the host cell cytosol (21). The subsequent transcriptional upregulation of genes in the TNF signaling pathway, as revealed by our RNA-seq analysis, indicates a robust macrophage activation. TNF-α is a master regulator of inflammation, and its overproduction is a hallmark of severe *V. vulnificus* sepsis, contributing to septic shock and organ failure (22). The concomitant activation of the IL-17 pathway is particularly intriguing, as this pathway is typically associated with neutrophilic inflammation and autoimmune pathology (23). Its induction by OMVs suggests a potential role in amplifying the inflammatory cascade, possibly through synergistic signaling with TNF-α or via the induction of other cytokines and chemokines that recruit and activate neutrophils, a key cell type in *V. vulnificus* lesion formation (24).

The most salient finding of this study is the direct link established between OMV-induced TNF signaling and mitochondrial damage. Mitochondria are not only cellular powerhouses but also central hubs for apoptosis, innate immune signaling and metabolic regulation (25). The observed mitochondrial damage in TEM and the dissipation of the MMP are classical indicators of mitochondrial dysfunction, often preceding the mitochondrial permeability transition and cytochrome c release, key events in the intrinsic apoptotic pathway (26). While bacterial pathogens and their components can directly target mitochondria (17, 27), our data with R-7050 treatment present a paradigm shift for *V. vulnificus* OMVs. The significant alleviation of both structural and functional mitochondrial damage upon TNFR inhibition unequivocally demonstrates that the key driver of this injury is the activation of TNF signaling pathway. TNFR1 engagement can activate multiple downstream pathways, including NF-κB and caspase-8. Crucially, activated caspase-8 can cleave and activate the BID protein to tBID, which translocates to mitochondria, inducing outer membrane permeabilization and the loss of MMP (28). Furthermore, TNF-α can also induce reactive oxygen species (ROS) production, which could exacerbate mitochondrial damage (29).

The biological implications of this OMV-TNF-mitochondria axis are profound. Firstly, damaged mitochondria can release potent DAMPs like mtDNA and formylated peptides, which can activate the NLRP3 inflammasome and cGAS-STING pathways (30, 31), thereby exacerbating the inflammatory response beyond the initial TNF signals. Secondly, severe mitochondrial dysfunction can lead to apoptotic or even pro-inflammatory necroptotic cell death of macrophages (32, 33). Alternatively, if the cells undergo necroptosis, the release of intracellular contents would further fuel inflammation (34). Finally, mitochondrial damage disrupts oxidative phosphorylation (35), potentially shifting macrophages toward a glycolytic, pro-inflammatory state, which sustains high TNF-α production. This creates a feed-forward loop where TNF damages mitochondria, and the dysfunctional metabolism perpetuates TNF synthesis. While the primary model in this study is a murine macrophage cell line, the validation in primary human macrophages or *in vivo* models would strengthen the clinical relevance. Although R-7050 is a specific tool, future studies using genetic approaches would provide definitive confirmation of our findings. The precise OMV components responsible for initiating the TNF response remains to be identified, and candidates include LPS, other TLR agonists, or pore-forming toxins that cause potassium efflux and inflammasome priming. Research has demonstrated that *V. vulnificus* cytolysin can stimulate macrophages to produce TNF-α (36). However, further investigation is required to ascertain whether this component is the principal factor responsible for the activation of the TNF pathway by its OMVs.

This work elucidates a novel pathogenic cascade for *V. vulnificus*, where shed OMVs act as a potent inflammatory trigger that co-opts the host's own TNF signaling machinery to inflict mitochondrial damage on macrophages. *V. vulnificus* uses OMVs to turns the host's defense mechanism against its own cellular infrastructure, representing a sophisticated form of immune subversion. This study elucidates, at least partially, the mechanisms underlying the cytokine storm and tissue damage associated with severe *V. vulnificus* infections. Targeting this pathway may offer a novel adjunctive therapeutic approach to mitigate excessive inflammation and enhance clinical outcomes in severe infection. Consequently, our findings contribute a substantial advancement to the understanding of OMV-mediated pathogenesis and underscore the mitochondrion as a pivotal site of host-pathogen interaction in the battle against *V. vulnificus*.

## Data Availability

The original contributions presented in the study are publicly available. This data can be found here: Supplemental sequencing data are available in the NCBI Sequence Read Archive (SRA) under BioProject accession number PRJNA1392675.
